# Highly sensitive electrochemical immunosensor based on methylene blue-reduced graphene oxide nanocomposites as signal probes for IL-6 detection in gingival crevicular fluid samples

**DOI:** 10.3389/fchem.2025.1549927

**Published:** 2025-04-02

**Authors:** Changfeng Zhu, Hongxin Wang, Jiyang Liu

**Affiliations:** ^1^ Department of Stomatology, Beijing Hospital of Integrated Traditional Chinese and Western Medicine, Beijing, China; ^2^ Key Laboratory of Surface & Interface Science of Polymer Materials of Zhejiang Province, Department of Chemistry, School of Chemistry and Chemical Engineering, Zhejiang Sci-Tech University, Hangzhou, China

**Keywords:** methylene blue, reduced graphene oxide, polydopamine, electrochemical immunosensor, IL-6

## Abstract

As an important inflammatory cytokine, interleukin-6 (IL-6) can mediate the entire pathological process of periodontitis and is closely associated with the degree of inflammation. Therefore, it is critical to develop convenient quantitative methods for monitoring IL-6 quantity in gingival crevicular fluid. In this study, methylene blue (MB)-decorated reduced graphene oxide (rGO) is employed as signal probe to further support the antibody-enabling specific recognition of IL-6. Due to π–π stacking and electrostatic interactions, rGO-MB nanocomposites can be stably obtained. rGO with good conductivity and large surface area characteristics promotes the redox signals of MB on the glassy carbon electrode (GCE). In addition, through the simple *in situ* self-polymerization of dopamine, the polydopamine (PDA) obtained can be not only directly used as a biological crosslinking agent for covalent immobilization of anti-IL-6 antibody but can also be regarded as a protective layer to enhance the stability of rGO-MB on the GCE surface. Such a designed PDA/rGO-MB/GCE-based immunosensor enables specific binding with IL-6 and produces a decreased electrochemical signal for MB, realizing the selective and sensitive quantitative measurement of IL-6. Consequently, our fabricated PDA/rGO-MB/GCE-based electrochemical immunosensor has an excellent linear relationship with IL-6 ranging from 1 pg/mL to 100 ng/mL, with a limit of detection as low as 0.48 pg/mL. Moreover, our as-prepared sensing strategy shows accurate monitoring of the IL-6 quantity in gingival crevicular fluid samples.

## 1 Introduction

Periodontal disease is a chronic nonspecific condition with a 70%–85% prevalence in China, making it a major cause of tooth loss in middle-aged and older adults. Clinical diagnosis mainly relies on periodontal examination indices and digital subtraction radiography. However, this diagnostic approach has certain delays, making it difficult to assess periodontal risk early and evaluate the activity of periodontal disease in a timely manner. With the continuous development of molecular biology and biological detection technologies, many researchers have begun to focus on the study of biomarkers in gingival crevicular fluid during the active phase of periodontitis. Interleukin-6 (IL-6) is a kind of important inflammatory cytokine which can mediate the entire pathological process of periodontitis and is closely associated with the degree of inflammation (Noh et al., 2013; Kardeşler et al., 2011). Therefore, developing simple, accurate and cost-effective analytical techniques to meet the demand of point-of-care testing in clinical detection is important.

Currently, enzyme-linked immunosorbent assay is the standard technique for the quantitative monitoring of IL-6 in clinical studies (Kingsmore, 2006). However, there are several disadvantages to this method—it is time-consuming and requires a large sample volume and complex operation. Electrochemical sensors offer a powerful analytical tool with numerous merits, including rapid response, high selectivity and sensitivity, cost-effectiveness, portability, facile operation, and easy miniaturization (Su et al., 2022; Huang L. et al., 2023; Zeng et al., 2023). These advantages make them invaluable for a wide range of applications in diverse fields (Zhou et al., 2024a; Wu et al., 2025; Fan et al., 2024). There have been many studies on electrochemical biosensors for the sensitive determination of IL-6 (McCrae et al., 2023; Zhang W. et al., 2023; Zhu et al., 2025). Recognition elements can be categorized as aptamer, antibody, and IL-6 receptor. Electrochemical immunosensors based on the high specific affinity between antibody and IL-6 have received growing attention, and it is highly desirable to design convenient electrochemical immunosensors for the sensitive determination of IL-6.

Electrochemical sensors based on immobilized electroactive probes on the surface of the sensing interface provides an attractive analytical strategy thanks to the reagent saving and simplified assay procedure, indicating it as a versatile biosensing platform for practical applications (Ma et al., 2024; Zhang et al., 2024; Li et al., 2023). Several organic dyes (e.g., methylene blue (MB) (Krishnan et al., 2023) and thionine (Ding et al., 2022)) or transition-metal complex ions (e.g., tris(hexa-ammineruthenium (III) (Huang Z. et al., 2023), potassium ferricyanide (Yan et al., 2022a; Zhang T. et al., 2023; Guo et al., 2023), 1,10-phenanthroline)iron (II) (Duan et al., 2024; Huang et al., 2024), tris(2,2-bipyridine)ruthenium (II) (Yang et al., 2022; Gao et al., 2025), and tris(1,10-phenanthroline)ruthenium (II) (Chen et al., 2022)) serving as redox indicators have been applied in the field of electroanalytical determination. Among these, MB molecules with positive charges possess water solubility, inherent redox property, and good reversibility. Moreover, the formal potential of MB molecules far exceeds that of common co-existing substances (e.g., uric acid and ascorbic acid) in biological samples, and it is suitable for practical sample analysis application. In comparison with MB molecules in solution, reagentless electrochemical immunosensors with MB molecules immobilized on the electrode surface show merits of sparing reagents and cost, convenient detection procedure, and reusability (Zhou et al., 2023; Chen et al., 2023). Graphene and its derivatives have shown great potential in the field of biosensors due to their unique properties such as high surface area, high catalytic activity, excellent electrical conductivity, and chemical stability (Zhang C. et al., 2023; Zou et al., 2022; Zhou et al., 2024b; Zhao et al., 2025; Zhou et al., 2022; Deng et al., 2021; Zhu et al., 2022). To increase electrode conductivity, reduced graphene oxide (rGO) (Chen et al., 2013) and carbon nanotubes (Zhang et al., 2022) have been employed to hybridize with MB molecules to accelerate the electron transfer procedure of MB on the sensing interfaces.

This study reported a simple electrochemical immunosensor for the sensitive determination of IL-6 based on the rGO-MB nanocomposite. The rGO-MB nanocomposite was obtained by simply mixing rGO and MB solution *vi*a π–π stacking and electrostatic adsorption interactions. Polydopamine self-polymerized from dopamine not only forms a uniform protective layer but also offers functional sites (dopaquinone) for grafting species bearing −SH or −NH_2_ moieties (Alfieri et al., 2022). Arising from the dual-functional property of PDA, rGO-MB nanocomposite can be stably modified on the supporting electrode surface (glassy carbon electrode—GCE), and antibody corresponding to IL-6 can then be covalently immobilized on the electrode. Such obtained rGO-MB/GCE-based immunosensor integrates the electrochemical probe (MB), electron transfer accelerator (rGO), and biological recognition element (antibody) into the sensing interface, realizing the reagentless electrochemical measurement of IL-6. Moreover, analysis of IL-6 in gingival crevicular fluid was tested by our fabricated immunosensor, offering a universal platform to detect a series of important biomarkers.

## 2 Experimental section

### 2.1 Chemicals and materials

Monolayered graphene oxide (GO) aqueous dispersion was provided from Hang-zhou Gaoxi Technology Co., Ltd. (Hangzhou, China) at a 1 mg/g concentration. Several inflammatory factors, including interleukin-1β (IL-1β), tumor necrosis factor-α (TNF-α), matrix metalloproteinase-9 (MMP-9), interferon-γ (IFN-γ), c-reactive protein (CRP), interleukin-6 (IL-6), and IL-specific antibody, were ordered from Beijing KEY-BIO Biotech Co., Ltd. (Beijing, China). Methylene blue trihydrate (MB), glucose (Glu, 100%), uric acid (UA), ascorbic acid (AA), 3-hydroxytyramine hydrochloride (DA), bovine serum albumin (BSA), potassium ferrocyanide (K_4_Fe(CN)_6_, 99.5%), potassium ferricyanide (K_3_Fe(CN)_6_, 99.5%), sodium hydroxide (NaOH, 97%), sodium chloride (NaCl), disodium hydrogen phosphate dodecahydrate (Na_2_HPO_4_·12H_2_O, 99%), potassium chloride (KCl, 99.5%), and sodium dihydrogen phosphate dihydrate (NaH_2_PO_4_·2H_2_O, 99%) were bought from Shanghai Aladdin Bio-Chem Technology Co., Ltd. (Shanghai, China). Hydrazine hydrate (80%) was obtained from Hangzhou Shuanglin Chemical Reagent Co., Ltd. (Hangzhou, China). Ammonia solution (25.0–28.0 wt%) was bought from Hangzhou Gaojing Fine Chemical Co., Ltd. (Hangzhou, China). Phosphate-buffered saline (PBS) was obtained by mixing NaH_2_PO_4_·2H_2_O and Na_2_HPO_4_·12H_2_O. Gingival crevicular fluid samples provided by a healthy man were received from the Beijing Hospital of Integrated Traditional Chinese and Western Medicine (Beijing, China). All aqueous solutions were prepared by ultrapure water (Mill-Q Sys-tems, Millipore, 18.2 MΩ cm) throughout the study.

### 2.2 Measurements and instrumentations

Ultraviolet–Vis (UV-Vis) absorption spectra were recorded on a UV-2450 UV-Vis spectrometer (Shimadzu, Japan). Scanning electron microscopy (SEM) measurements were carried out with a GeminiSEM 360 (Germany, ZEISS) scanning emission electron microscope. X-ray photoelectron spectroscopy (XPS) analyses were performed on a PHI5300 electron spectrometer (PE Ltd., United States) with Mg Kα radiation of 250 W and 14 kV.

A PGSTAT302N Autolab electrochemical workstation (Metrohm Autolab, Switzerland) was used to conduct cyclic voltammetry (CV), electrochemical impedance spectroscopy (EIS), and differential pulse voltammetry (DPV) measurements. Bare or modified GCE, an Ag/AgCl, and a platinum wire were regarded as the working, reference, and auxiliary electrodes, respectively. The experimental parameters for EIS measurements are: frequency range (0.1 Hz–100 kHz) and perturbation amplitude (5 mV).

### 2.3 Fabrication of the rGO-MB/GCE electrode

Reduced graphene oxide (abbreviated as rGO) nanosheets were synthesized as per Li et al. (2008). Briefly, 32 mL H_2_O, hydrazine solution (7.0 μL, 80 wt%), and 120 μL ammonia solution (25–28 wt%) were added to a round-bottom flask containing GO dispersion solution (8 mL, 1 mg/mL) and then stirred in a water bath at 85°C for 3 h. Subsequently, stable rGO dispersion was obtained after centrifugation at 3,000 rpm for 30 min.

MB-decorated rGO nanocomposite (MB-rGO) was synthesized by mixing rGO (850 μL, 0.2 mg/mL) and MB (150 μL, 0.2 mg/mL), and the dispersion was then subjected to ultrasonic treatment for 30 min. After centrifugation at 12,000 rpm three times for 10 min each time to exclude the supernatant, the precipitate was collected—rGO-MB hybrid. Finally, the precipitate was redispersed in 500 μL ultrapure water to obtain an rGO-MB solution.

GCE was polished using different specifications of alumina powder (0.5 μm, 0.3 μm, and 0.05 μm) in sequence. Next, GCE was immersed in anhydrous ethanol and ultrapure water alternatively under ultrasonication. After cleaning with a large amount of water, GCE was coated by rGO-MB complex solution (10 μL) and further dried at 60°C to finally achieve GCE modified with rGO-MB—“rGO-MB/GCE”.

### 2.4 Fabrication of a PDA/rGO-MB/GCE-based sensor

The rGO-MB/GCE modified with a polydopamine (PDA) layer (PDA/rGO-MB/GCE) was obtained by immersing rGO-MB/GCE in a 0.1 M PBS (pH = 8.5) containing 0.38 mg/mL DA solution was then washed with ultrapure water to exclude the redundant DA on the sensing interface. We then coated 10 μL antibody possessing high affinity to IL-6 (10 μg/mL, dispersed in a 0.01 M PBS (pH 7.4)) on the PDA/rGO-MB/GCE for incubation at 4°C for 60 min. Subsequently, the electrode obtained was rinsed with 0.01 M PBS (pH = 7.4) several times and coated with 1 wt% BSA at 4°C for 30 min to avoid nonspecific adsorption. Ultimately, the immunosensing interface was designated as the “BSA/Ab/PDA/rGO-MB/GCE”, which was used to quantitatively analyze IL-6 amounts.

### 2.5 Electrochemical test procedure for target IL-6

IL-6 at various concentrations was subsequently coated on the BSA/Ab/PDA/rGO-MB/GCE surface and incubated at 4°C for 60 min. Then, BSA/Ab/PDA/rGO-MB/GCE after binding with target IL-6 was placed into a 0.1 M PBS (pH = 7.4) solution and tested by electrochemical techniques. For the analysis of IL-6 in gingival crevicular fluid samples, the samples directly received were diluted with 0.01 M PBS (pH = 7.4, dilution ratio is 100) and spiked with several known concentrations of IL-6. Our fabricated BSA/Ab/PDA/rGO-MB/GCE sensor was employed to test the above real samples, and the test results were compared with the known concentrations of IL-6.

## 3 Results and discussion

### 3.1 Fabrication of BSA/Ab/PDA/rGO-MB/GCE immunosensor and its mechanism for the electrochemical detection of IL-6

**SCHEME 1:**
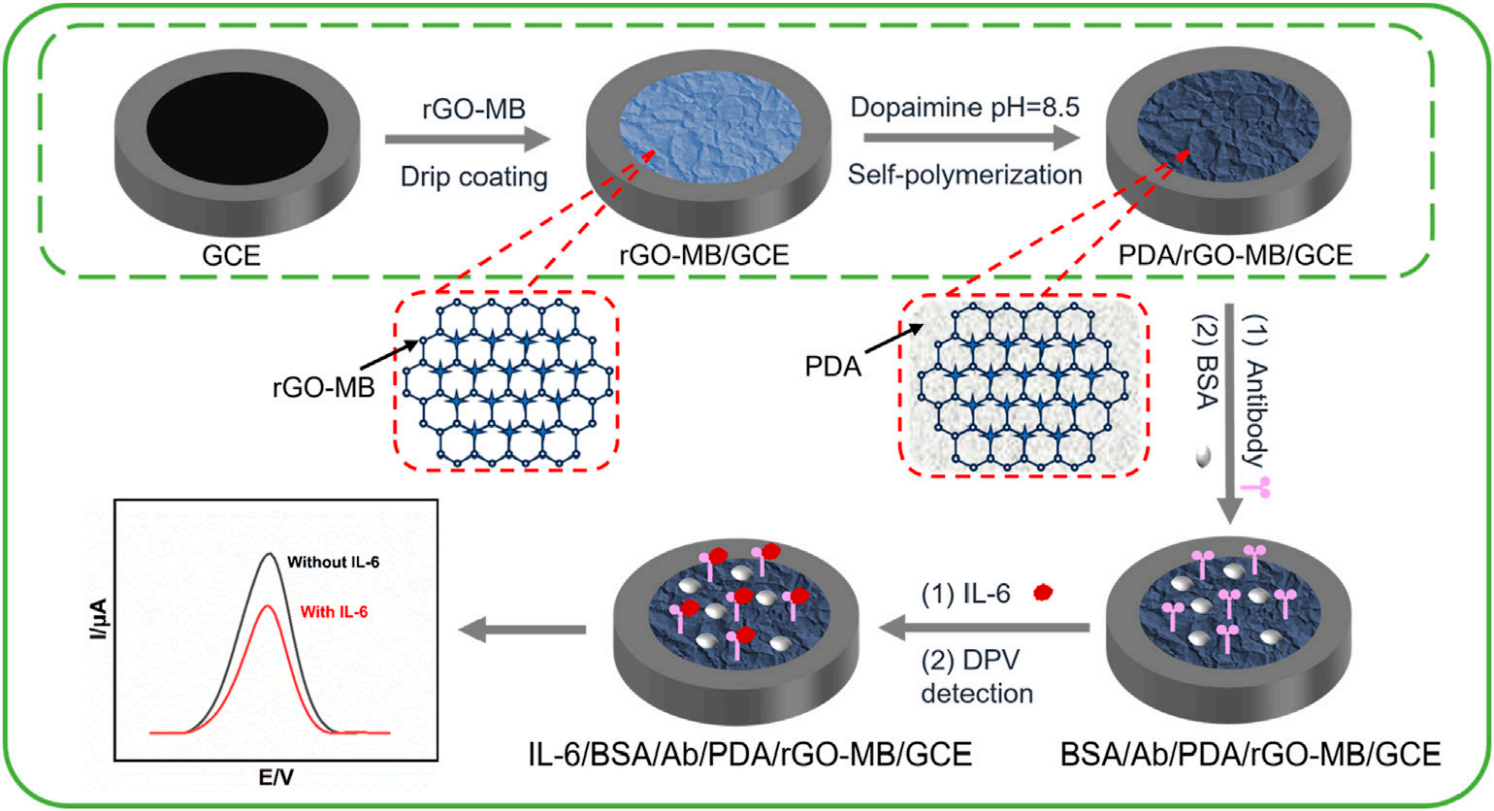
Fabrication procedure of the BSA/Ab/PDA/rGO-MB/GCE immunosensor and the electrochemical determination of IL-6.

Scheme 1 illustrates the fabricated procedure of the BSA/Ab/PDA/rGO-MB/GCE immunosensor and its mechanism for the electrochemical determination of IL-6. Both rGO and MB bear the π-conjugated structure and carry the opposite charges (negative charges for rGO and positive charges for MB; the *pK*_a_ of MB is 11.6) in the tested condition (pH 7.4). rGO-MB nanocomposite can be prepared by simply mixing rGO dispersion solution with MB solution *via* π–π stacking and electrostatic interactions, which is subsequently dropped onto the clean GCE surface to obtain rGO-MB/GCE. rGO with the characteristics of good conductivity and large surface area promotes the redox signals of MB on the GCE surface. Then, PDA self-polymerized from DA under weak alkaline conditions can directly form a dense and adherent layer on the surface of rGO-MB/GCE, which not only acts as the protective layer for avoiding the leakage of the inner rGO-MB layer but also provides functional sites for the further modification of anti-IL-6 antibody. Note that −SH or −NH_2_ groups on the anti-IL-6 antibody can react with many functional groups (e.g., catechol, amine) on PDA through Schiff-based or Michael addition reactions, which enable the development of highly specific and sensitive biosensors. After avoiding non-specific adsorption using BSA, the electrochemical immunosensing interface is achieved, denoted “BSA/Ab/PDA/rGO-MB/GCE”. Arising from the specific immunoaffinity towards target IL-6, IL-6 can be captured at the BSA/Ab/PDA/rGO-MB/GCE and hinders the electron transfer procedure of immobilized MB molecules, resulting in a decline in electrochemical signals. Therefore, the decreased electrochemical signals exhibit a quantitative relationship with the IL-6 concentration, enabling the accurate monitoring of IL-6 quantity.

### 3.2 Characterizations of rGO-MB nanocomposites

**FIGURE 1:**
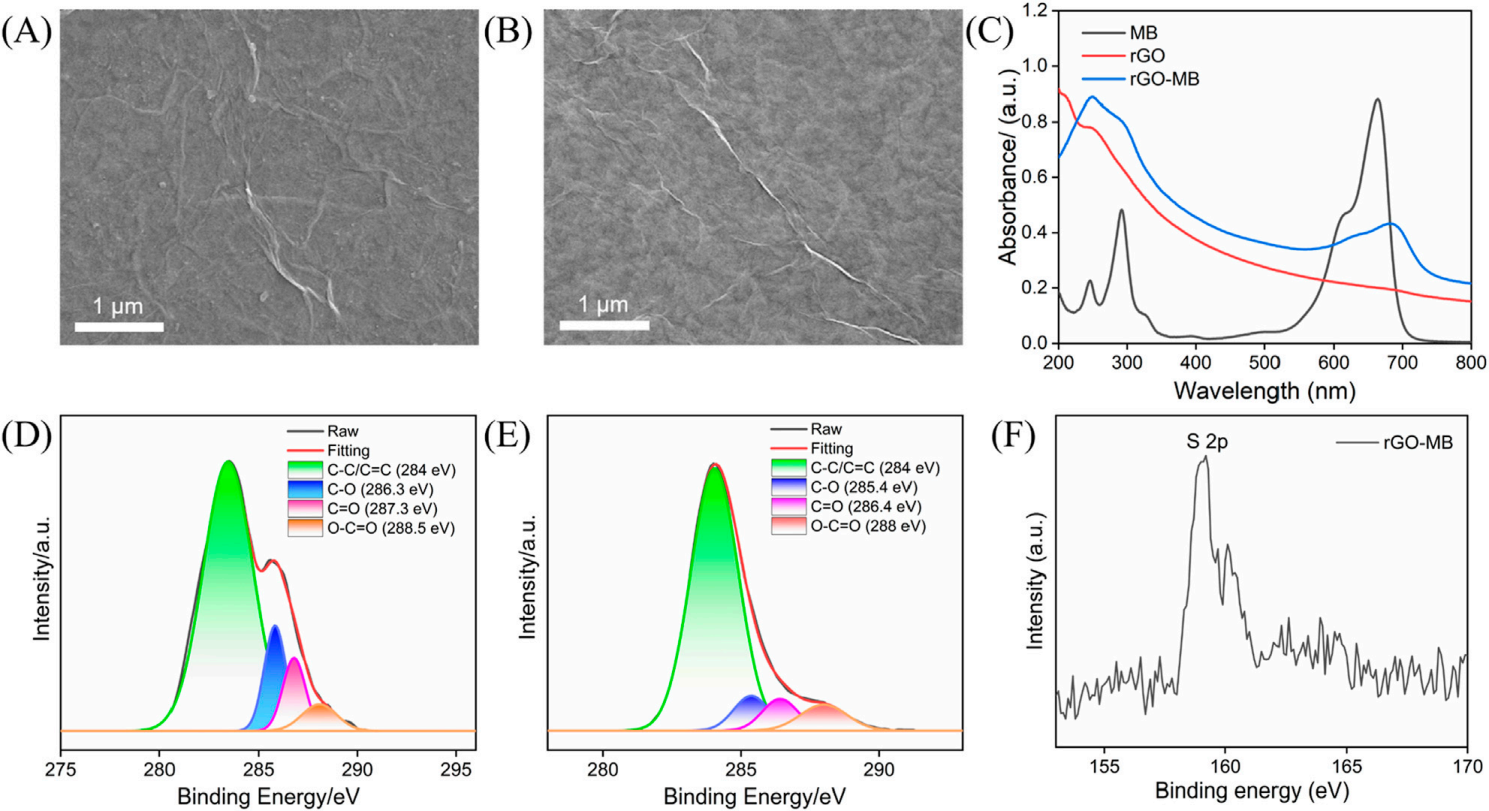
Characterizations of rGO-MB: **(A,B)** SEM, rGO **(A)** and rGO-MB nanocomposite **(B)**; **(C)** UV–Vis; **(D–F)** XPS, C1s spectra of GO **(D)** and rGO-MB **(E)**, and S 2p XPS spectrum of r GO-MB **(F)**.

To confirm the successful preparation of rGO-MB nanocomposite, techniques including scanning electron microscopy (SEM), UV–visible spectroscopy (UV-vis), and X-ray photoelectron spectroscopy (XPS) were performed. As shown in Figures 1A and B, rGO has some wrinkles, and the introduction of MB does not affect the structure of rGO. As shown in Figure 1C, rGO has a weak adsorption peak at 253 nm in UV–vis spectrum, which is attributed to the π–π* transition of C–C/C=C bonds in the aromatic structure of rGO (red curve) (Chang et al., 2022; Yan et al., 2022b). The strong adsorption peak at 664 nm shown in MB arises from the π–π* transition of aromatic structure in the MB monomer (black curve). In contrast, rGO-MB nanocomposite remains the adsorption peak at 253 nm of rGO and shifts the prominent absorption peak at 664 nm of MB to 685 nm, implying that the MB and rGO planes are close and further verifying the successful preparation of rGO-MB nanocomposite. By comparing the C1s XPS spectra of GO and rGO-MB nanocomposite (Figures 1D, E), the intensities of oxygen-containing groups (C–O, C=O and O–C=O), remarkably decline, suggesting the presence of rGO in the rGO-MB nanocomposite (Lv et al., 2022; Cui et al., 2023; Zheng et al., 2022). In addition, rGO-MB shows the typical characteristic peak of S 2p (Figure 1F), which is the S element in MB molecules. Based on the above data, the hybrid material consisting of rGO and MB (rGO-MB) nanocomposite was successfully prepared.

**FIGURE 2:**
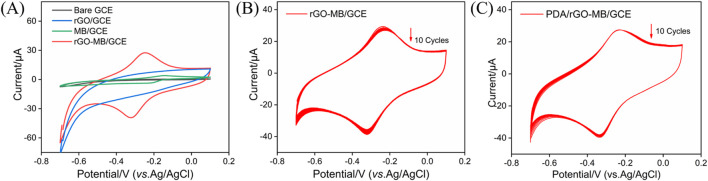
**(A)** CV plots of bare GCE, rGO/GCE, MB/GCE, and rGO-MB/GCE in 0.1 M PBS (pH = 7.4). Continuous CV responses of rGO-MB/GCE **(B)** and PDA/rGO-MB/GCE **(C)** in 0.1 M PBS (pH = 7.4). Scan rate: 50 mV/s.

**FIGURE 3:**
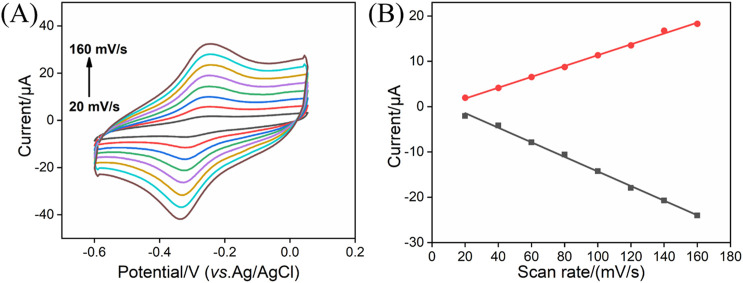
**(A)** CV curves of PDA/rGO-MB/GCE in 0.1 M PBS (pH = 7.4) at different scan rates (20–160 mV/s). **(B)** Relationship between redox peak currents and scan rate.

After dropping the rGO-MB nanocomposite onto the electrode surface, the electrochemical behavior of the rGO-MB/GCE surface was studied by cyclic voltammetry (CV). Figure 2A depicts the CV curves of bare GCE, rGO/GCE, MB/GCE, and rGO-MB/GCE in buffer solution. No redox peak was found at either the bare GCE or rGO/GCE. Moreover, the capacity current of rGO/GCE is apparently larger than that of bare GCE, which is due to the larger active surface area of rGO. A pair of redox peaks is displayed at the GCE modified with MB molecules (MB/GCE), with a formal potential of −0.183 V (anodic peak potential (*E*_pa_) at −0.148 V and cathodic peak potential (*E*_pc_) at −0.217 V), which is assigned to the electrochemical reaction procedure of MB molecules. In contrast, the co-existing rGO with MB is able to accelerate the electron transfer of MB on the GCE, giving rise to a couple of well-defined redox peaks at the MB-rGO/GCE and decreased formal potential (−0.289 V, *E*_pa_ at −0.250 V and E_pc_ at −0.328 V). To verify the stabilization effect of the PDA layer for inner rGO-MB nanocomposite, rGO/GCE and PDA/rGO-MB/GCE were successively scanned in buffer solution for ten cycles. As shown in Figures 2B and C, both the anodic (*I*_pa_) and cathodic (*I*_pc_) peak current of MB molecules at the rGO-MB/GCE gradually decrease with the increasing scanning cycles, whereas those at the PDA/rGO-MB/GCE remain unchanged, demonstrating the excellent stability of PDA/rGO-MB/GCE due to the PDA layer. In addition, the effect of the scan rate on the redox peak currents at the PDA/rGO-MB/GCE was recorded by CV, with the results displayed in Figure 3. It can be determined that redox peak currents of MB molecules at the PDA/rGO-MB/GCE are enhanced with an increase of scan rate in the range of 20 mV/s to 160 mV/s. The obtained *I*_pa_ or *I*_pc_ displays good linear relationship with scan rate (*v*), yielding two linear fitting equations of *I*_pa_ (μA) = 0.120 *v* (mV/s) −0.608 (*R* ^2^ = 0.997) and *I*_pc_ (μA) = −0.161 *v* (mV/s) + 1.77 (*R* ^2^ = 0.997). This indicates that the electrochemical reaction of immobilized MB at the PDA/rGO-MB/GCE is surface-controlled.

### 3.3 Feasibility of the constructed BSA/Ab/PDA/rGO-MB/GCE immunosensor

**FIGURE 4:**
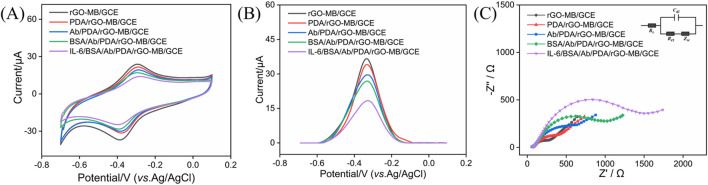
CV **(A)** and DPV plots **(B)** of rGO-MB/GCE, PDA/rGO-MB/GCE, Ab/PDA/rGO-MB/GCE, and BSA/Ab/PDA/rGO-MB/GCE before and after testing with 1 ng/mL IL-6 in 0.1 M PBS (pH = 7.4). **(C)** EIS curves of different electrodes tested in 0.1 M KCl containing 2.5 mM Fe(CN)_6_^3−/4−^. The inset in **(C)** is an equivalent circuit, including electron transfer resistance (*R*_ct_), two-layer capacitance (*C*_dl_), solution resistance (*R*_s_), and Warburg impedance (*Z*_w_).

Stepwise fabrication of the BSA/Ab/PDA/rGO-MB/GCE immunosensor and its feasibility for IL-6 detection was studied by CV, DPV, and EIS. Figures 4A and B show the CV (a) and DPV (b) of rGO-MB/GCE, PDA/rGO-MB/GCE, Ab/PDA/rGO-MB/GCE, and BSA/Ab/PDA/rGO-MB/GCE before and after testing 1 ng/mL IL-6 in detection solution. As is evident, electrochemical responses at the rGO-MB/GCE decrease after consecutive modification of PDA, anti-IL-6 antibody (Ab), and BSA thanks to their poor conductivity impeding electron transfer at the sensing interface. Upon incubation with 1 ng/mL target IL-6, a remarkably reduced electrochemical signal is observed at the BSA/Ab/PDA/rGO-MB/GCE sensor, suggesting the potential of our developed biosensor for monitoring IL-6 amounts. In addition, the constructed immunosensor was verified by EIS in Figure 4C. As seen, the semicircle diameter in the high-frequency region corresponding to the charge transfer resistance at the electrode/electrolyte interface progressively increases with the stepwise modification procedure and IL-6 detection.

### 3.4 Optimum conditions for the detection of IL-6 and sensitive detection of IL-6 using BSA/Ab/PDA/rGO-MB/GCE

**FIGURE 5:**
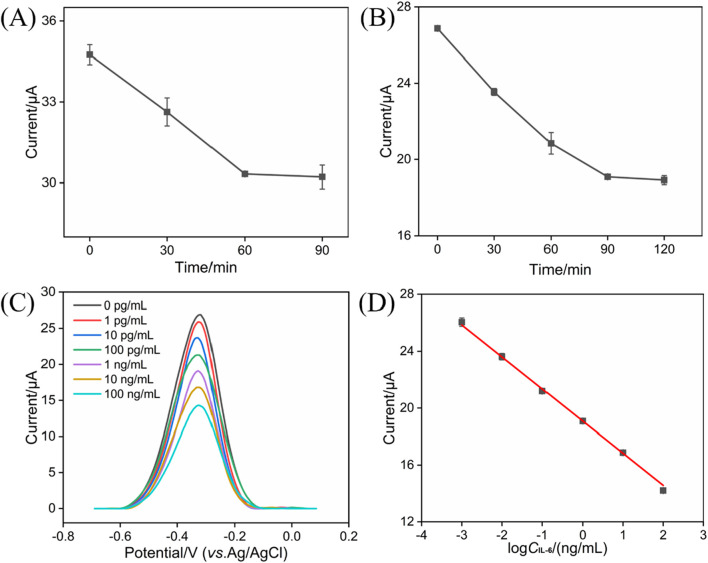
**(A)** Effect of incubation time between IL-6-specific antibody and the PDA/rGO-MB/GCE on the current response. The concentration of anti-IL-6 antibody is 10 μg/mL. **(B)** Effect of incubation time between the BSA/Ab/PDA/rGO-MB/GCE and target IL-6 on the obtained current response. The concentration of IL-6 is 1 ng/mL. **(C)** DPV curves of the BSA/Ab/PDA/rGO-MB/GCE immunosensor to various concentrations of IL-6 (1 pg/mL, 10 pg/mL, 100 pg/mL, 1 ng/mL, 10 ng/mL, and 100 ng/mL). **(D)** Corresponding calibration curve of IL-6 detection. Error bars in **(A, B, D)** are standard deviations (SDs) of three measurements.

Prior to the IL-6 detection, the incubation times of anti-IL-6 antibody and target IL-6 were studied, with the results shown in Figures 5A and B. As is evident, electrochemical response can be unchanged after 60 and 90 min, respectively. Therefore, 60 and 90 min were selected as the optimal incubation time for anti-IL-6 antibody and target IL-6, respectively. Figure 5C shows the representative DPV curves of the BSA/Ab/PDA/rGO-MB/GCE immunosensor before and after incubation with various concentrations of IL-6 (1 pg/mL, 10 pg/mL, 100 pg/mL, 1 ng/mL, 10 ng/mL, and 100 ng/mL). As is evident, the obtained anodic peak current (*I*) decreases gradually with the increasing concentration of IL-6 (*C*_IL-6_). According to the DPV results shown in Figure 5C, a good fitting linear relationship between the I and the logarithm of IL-6 concentration was achieved, yielding a regression equation of *I* (μA) = −2.26 log *C*_IL-6_ (ng/mL) + 19.1 with *R* ^2^ of 0.997 (Figure 5D). The limit of detection (LOD) is calculated by substituting the minimum acceptable signal (*X*_L_) into the above linear equation. *X*_L_ can be obtained using *X*_L_ = *X*_b_ + *3S*_b_, where *X*_b_ denotes the average response signal to the blank solution, with *S*_b_ as the standard deviation of its 11 time-measured signals. Based on the above method, LOD achieved at the BSA/Ab/PDA/rGO-MB/GCE sensor is 0.48 pg/mL. Table 1 compares the analytical performance—including electrochemical technique, linear range, and LOD of our fabricated BSA/Ab/PDA/rGO-MB/GCE immunosensor—with other electrochemical immunosensors. As seen, our fabricated BSA/Ab/PDA/rGO-MB/GCE immunosensor has a comparable wide linear range and low LOD. In addition, our as-prepared immunosensor integrates the electroactive probe (MB) into the sensing interface, which spares the addition of a probe into the detection solution and demonstrates the convenience of the operation.

**TABLE 1:** Comparison of detection performance of different reported electrochemical strategies based on the immunocomplex reaction for monitoring IL-6 amounts.

Electrode	Technique	Linear range (ng/mL)	LOD (pg/mL)	Ref.
BSA/Ab/AuNPs@THI/GCE	DPV	0.01–100	1.85	Wang et al. (2023)
IL-6/MCH/Apt/AuNPs/PATP/PABA/GCE	EIS	0.005–100	1.6	Tertis et al. (2019)
Ab2/IL-6/PVA/Ab1/EDC-NHS/SPE	SWV	0.002–0.25	0.78	Cancelliere et al. (2023)
BSA/Ab/APTES/Cu/ZnO/GS	DPV	0.001–100	0.43	Lakshmi Narayanan et al. (2024)
BSA/Ab/MUA-MPA/AuNPs/AgNWs/CNT-PDMS	DPV	0.001–100	0.322	Li et al. (2025)
BSA/Ab/AuNPs@NMC/GCE	DPV	0.0005–1.2	0.14	Liu et al. (2021)
BSA/Ab/Sulfo-LC-SPDP/SPGE	DPV	0.001–0.02	0.088	Cruz et al. (2022)
BSA/Ab/ERGO-AuPdNPs/HCPE	LSV	0.0001–100	0.059	Lou et al. (2014)
BSA/Ab/PDA/rGO-MB/GCE	DPV	0.001–100	0.48	This work

AuNPs@THI: Au nanosphere–thionine composites; MCH: mercaptohexanol; PATP: p-aminothiophenol; PABA: p-aminobenzoic acid; PVA: polyvinyl alcohol; EDC-NHS: N-(3-dimethylaminopropyl)-N′-ethyl carbodiimide and N-hydroxysuccinimide; SPE: screen-printed electrode; SWV: square-wave voltammetry; APTES: 3-aminopropyltriethoxysilane; GS: graphite sheet; MUA-MPA: 11-mercaptoundecanoic acid and 3-mercaptopropionic acid; AgNWs: silver nanowires; CNT-PDMS: half-cured carbon nanotubes/polydimethylsiloxane; AuNPs@NMC: nanoporous mesoporous-carbon composite decorated with gold nanoparticles; Sulfo-LC-SPDP: sulfosuccinimidyl 6-(30-(2-pyridyldithio)propionamido) hexanoate; SPGE: screen-printed gold electrode; ERGO-AuPdNPs: nanocomposite of electrochemically reduced graphene oxide and gold palladium bimetallic nanoparticles; HCPE: electrically heated carbon electrode; LSV: linear sweep voltammetry.

### 3.5 Selectivity, repeatability, and stability of immunosensors

**FIGURE 6:**
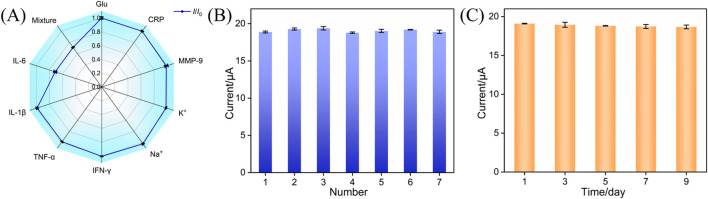
**(A)** Anodic peak currents tested at the proposed BSA/Ab/PDA/rGO-MB/GCE immunosensor before (*I*_0_) and after (*I*) testing with ten-fold concentration of Glu, CRP, MMP-9, K^+^, Na^+^, IFN-γ, TNF-α, IL-1β, IL-6 (1 ng/mL), or their mixture. **(B)** Anodic peak currents obtained on different BSA/Ab/PDA/rGO-MB/GCE immunosensors fabricated in parallel after incubation with 1 ng/mL IL-6. **(C)** Anodic peak currents obtained at the developed BSA/Ab/PDA/rGO-MB/GCE immunosensor to 1 ng/mL IL-6 after storage for different days. The error bars in **(A–C)** are the SD of three measurements and the tested solution is 0.1 M PBS (pH = 7.4).

Parameters such as selectivity, repeatability, and stability are very important for evaluating electrochemical sensors. Several co-existing substances which may interfere with IL-6 detection in real samples (e.g., gingival crevicular fluid) were incubated with the BSA/Ab/PDA/rGO-MB/GCE immunosensor and tested by the DPV technique. As shown in Figure 6A, glucose (Glu), K^+^, Na^+^, c-reactive protein (CRP), interferon-γ (IFN-γ), interleukin-1β (IL-1β), tumor necrosis factor-α (TNF-α), and matrix metalloproteinase-9 (MMP-9) at the ten-fold concentration of IL-6 show negligible influence on the anodic peak current variation, suggesting the excellent selectivity of the immunosensor design. In addition, a mixture of the above interfering species and target IL-6 results in the comparable electrochemical signal variation with IL-6 at the BSA/Ab/PDA/rGO-MB/GCE immunosensor, demonstrating the satisfactory anti-interference ability of our proposed immunosensor. Figure 6B shows the anodic peak currents obtained at the seven BSA/Ab/PDA/rGO-MB/GCE immunosensors fabricated in parallel after incubation with 1 ng/mL IL-6. As shown, a rather low RSD of 1.2% is achieved, indicating the good repeatability of our proposed immunosensor. The stability of the fabricated BSA/Ab/PDA/rGO-MB/GCE immunosensor was evaluated by testing the anodic peak current of BSA/Ab/PDA/rGO-MB/GCE immunosensor after incubation with 1 ng/mL IL-6 over a 9-day storage at 4 °C. As seen in Figure 6C, the rather stable current response with an RSD value of 0.9% implies the excellent stability of the fabricated immunosensor.

### 3.6 Detection of IL-6 in gingival crevicular fluid samples

Table 2 lists the tested results in gingival crevicular fluid samples by our as-prepared BSA/Ab/PDA/rGO-MB/GCE and the known spiked concentrations of IL-6. It can be found that the addition of standard concentrations of IL-6 (0.0100 ng/mL, 1.00 ng/mL and 100 ng/mL) in gingival crevicular fluid samples of a healthy human can be accurately detected by the BSA/Ab/PDA/rGO-MB/GCE with acceptable RSD values (<1.3%) and recoveries (97.3–99.3%). The above data indicate the capacity of BSA/Ab/PDA/rGO-MB/GCE for monitoring IL-6 amount in gingival crevicular fluid samples.

**TABLE 2:** Determination of IL-6 in the gingival crevicular fluid.

Sample^a^	Spiked (ng/mL)^b^	Found (ng/mL)^b^	RSD (%)	Recovery (%)
Gingival crevicular fluid^a^	0.0100	0.00993	1.0	99.3
	1.00	0.986	0.6	98.6
	100	97.3	1.3	97.3

^a^ Gingival crevicular fluid diluted using PBS (0.01 M, pH 7.4) with dilution ratio of 100.

^b^ Concentration of IL-6 shown in table was obtained after dilution.

## 4 Conclusion

We developed a simple electrochemical immunosensor for the highly sensitive determination of IL-6 detection based on the electrochemical signal of immobilized MB-rGO nanocomposite and the immunoaffinity of anti-IL-6 antibody. The MB-rGO nanocomposite can be obtained by simply mixing MB molecules and rGO solution via π–π stacking and electrostatic interactions. The large surface area and good conductivity of rGO promote the redox signal of MB molecules, leading to an amplified electrochemical signal of immobilized MB-rGO nanocomposite on the GCE surface. Functionalization of PDA at the designed MB-rGO/GCE not only improves the stability of the electrochemical signal but also provides active sites for further immobilizing the anti-IL-6 antibody. When an MB-rGO/GCE-based immunosensor is incubated with target IL-6, an immunocomplex is formed at the sensing interface and leads to the decreased electrochemical signal of immobilized MB molecules, enabling the sensitive determination of IL-6. The proposed MB-rGO/GCE-based immunosensor with excellent selectivity, repeatability, and stability is capable of detecting IL-6 in gingival crevicular fluid samples, providing a universal probe-integrated electrochemical immunoplatform for a broad range of important biomarkers.

## Data Availability

The original contributions presented in the study are included in the article/supplementary material; further inquiries can be directed to the corresponding author.
